# Reply to Letter to the Editor

**DOI:** 10.4274/tjo.galenos.2020.11455

**Published:** 2020-12-29

**Authors:** Kadircan H. Keskinbora, Fatih Güven

**Affiliations:** 1 Bahçeşehir University Faculty of Medicine, Department of Ophthalmology; Bahçeşehir University Faculty of Medicine, Department of Medical Ethics and History of Medicine, İstanbul, Turkey; 2 University of Health Sciences Turkey, Bakırköy Training and Research Hospital, Clinic of Ophthalmology, İstanbul, Turkey

**Keywords:** Artificial intelligence, machine learning, ophthalmology, medical ethics

## Dear Editor,

First of all, we thank the author(s) for evaluating our article.^1^ As the author(s) pointed out, we emphasized in our article that artificial intelligence will be of great usefulness for screening and rapid diagnosis in ophthalmology.

Artificial intelligence (AI) is divided into 3 groups based on capability: 1) Weak/Narrow/Simple AI (ANI), 2) Strong/General AI (AGI), and 3) Artificial Super Intelligence (ASI).^2^ Here, possible ethical problems are solved by respecting the privacy of personal information and features that should be considered in the anonymization of information. Therefore, the serious ethical concerns regarding artificial intelligence are not related to the classification of massive photo data using simple/narrow artificial intelligence (ANI).

Medicine is a prominent field that has witnessed a nanotechnological revolution. However, due to the current views in philosophy and ethics, this emerging technology can be considered inconsistent or conflicting with what most ethicists in the area of medicine hold to be true. Nanotechnology and neuroscience are raising unavoidable questions concerning the ethical justification of human enhancement and intervention.^3^

The main ethical issues are related to the use of AI in general (AGI) and superintelligence (ASI) in particular. These two groups of AI need to be audited during their advancement, as they have the capacity to develop in a versatile and unpredictable direction. Providing safety measures to prevent any direct or indirect coercion can only be possible through continuous ethical evaluations and monitoring of technological development.^4^
